# Deep Pressure Sensitivity as a Sensory Marker in Autism Spectrum Disorder: Evidence from a Binational Pediatric Study

**DOI:** 10.1192/j.eurpsy.2026.11179

**Published:** 2026-07-31

**Authors:** M. T. Sindelar

**Affiliations:** Psychology, Unisal, Bahia Blanca, Argentina

## Abstract

**Introduction:**

Sensory alterations are explicitly recognized in DSM-5 as a part of the diagnostic criteria for Autism Spectrum Disorder(ASD). Among these, hyper-or hyporeactivity to sensory input and unusual interests in sensory aspects of the environment are highlighted. While atypical tactile responses are frequently reported, research on deep pressure sensitivity remains scarce, despite its potential clinical and developmental relevance.

**Objectives:**

This study aimed to evaluate differences in pressure sensitivity between children with ASD and neurodevelopmental peers, and to examine the feasibility of usinga simple,lowcost clinical tool for routine assessment.

**Methods:**

A total of 526 children participated (186 with ASD, 340 neurotypical) aged £-14 years, recruited from southern Argentina and Southern Italy.

Pressure sensitivity was measured with a pediatric-adapted aneroid sphygmomanometer. Controlled pressure was applied to Different body areas, including arms and legs, and perceptual thresholds were recorded through both verbal reports and observable behavioral responses. Group comparisons were conducted to test the hypothesis that children with ASD would require higher pressure values, expressed in millimeters of mercury(mmHg), to register the stimulus.

**Results:**

Children with ASD consistently demonstrated significantly higher pressure thresholds than neurotypical peers, confirming reduced sensitivity to deep tactile input (hyporeactivity). These findings were robust across sites and age groups, reinforcing their reliability and cross-cultural validity.

**Conclusions:**

This study provides novel empirical evidence that deep pressure hyporeactivity is a distinctive and measurable sensory feature of ASD.

The use of an accessible, inexpensive instrument highlights the feasibility of integrating this procedure into everyday clinical practice, especially in low-resource contexts. Beyond clinical implications, the findings may inform early screening protocols, support the design of individualized interventions in educational and therapeutic settings, and guide public health strategies addressing sensory dimensions of ASD.

**Disclosure of Interest:**

None Declared

